# Field Evaluation of PIMA Point-of-Care CD4 Testing in Rakai, Uganda

**DOI:** 10.1371/journal.pone.0088928

**Published:** 2014-03-10

**Authors:** Ronald M. Galiwango, Lawrence Lubyayi, Richard Musoke, Sarah Kalibbala, Martin Buwembo, Jjingo Kasule, David Serwadda, Ronald H. Gray, Steven J. Reynolds, Larry W. Chang

**Affiliations:** 1 Rakai Health Sciences Program, Kalisizo, Uganda; 2 Department of Epidemiology, Johns Hopkins Bloomberg School of Public Health, Baltimore, Maryland, United States of America; 3 Laboratory of Immunoregulation, Division of Intramural Research, National Institute for Allergy and Infectious Diseases, National Institutes of Health, Bethesda, Maryland, United States of America; 4 Division of Infectious Diseases, Department of Medicine, Johns Hopkins University School of Medicine, Baltimore, Maryland, United States of America; 5 School of Public Health, Makerere University College of Health Sciences, Kampala, Uganda; Fundacion Huesped, Argentina

## Abstract

**Objective:**

To assess the accuracy of PIMA Point-of-Care (POC) CD4 testing in rural Rakai, Uganda.

**Methods:**

903 HIV positive persons attending field clinics provided a venous blood sample assessed on site using PIMA analyzers per manufacturer's specifications. The venous samples were then run on FACSCalibur flow cytometry at a central facility. The Bland–Altman method was used to estimate mean bias and 95% limits of agreement (LOA). Sensitivity, specificity, negative predictive value (NPV), and positive predictive value (PPV) were calculated for a CD4 threshold of <350 and <500 cells/uL for antiretroviral eligibility.

**Results:**

There was a high correlation between PIMA and FACSCalibur CD4 counts (r = 0.943, p<0.001). Relative to FACSCalibur, the PIMA POC CD4 had negative mean bias of −34.6 cells/uL (95% LOA: −219.8 to 150.6) overall. The dispersion at CD4<350 cells/uL was 5.1 cells/uL (95% LOA: −126.6 to 136.8). For a threshold of CD4<350 cells/uL, PIMA venous blood had a sensitivity of 88.6% (95%CI 84.8–92.4%), specificity of 87.5% (95%CI 84.9–90.1%), NPV of 94.9% (95%CI 93.1–96.7%), and PPV of 74.4% (95%CI 69.6–79.2%). PIMA sensitivity and PPV significantly increased to 96.1% and 88.3% respectively with increased threshold of 500 cells/uL.

**Conclusions:**

Overall, PIMA POC CD4 counts demonstrated negative bias compared to FACSCalibur. PIMA POC sensitivity improved significantly at a higher CD4 threshold of 500 than a 350 cells/uL threshold.

## Introduction

Assessment of eligibility for antiretroviral therapy (ART) in low and middle-income countries (LMIC) is dependent on CD4 T-lymphocyte enumeration and, if unavailable, WHO clinical staging. [1], [2]. Additionally, CD4 testing is frequently used to monitor patients on ART. In most clinical settings, CD4 enumeration has typically used flow cytometry which has excellent precision, accuracy, and reproducibility. However, flow cytometry requires fresh venous blood and subsequent processing is often at a central facility distant from the sample collection point. Consequently, there is a significant time lapse from sample procurement to return of results, with delays in decisions on ART eligibility and initiation. These delays are a barrier to care and can result in losses to follow-up with negative patient and programmatic outcomes [3]–[6].

Point-of-care (POC) CD4 T-lymphocyte enumeration is a promising technology that could mitigate many of the barriers and delays associated with flow cytometry and improve access to care. POC CD4 testing could allow timely return of CD4 results to providers and patients, obviating the need for repeated patient visits and rapidly linking patients into care. Several evaluations of POC CD4 technology have been reported, but results have not been entirely consistent, and study settings have been largely confined to central laboratories in urban locations [7]–[13]. Important questions remain about how this technology compares to established methods in primary healthcare settings. We conducted a field evaluation of the PIMA POC CD4 test system (Alere Inc., Waltham, MA) in a rural setting in Rakai, Uganda.

## Materials and Methods

### Study Population

Rakai district in southwestern Uganda has a population of approximately half a million in a rural area of about 5,000 square kilometers. The Rakai Health Sciences Program (RHSP) is a community-based research organization with a focus on HIV/AIDS and reproductive health [14] which since 2004 has been a major PEPFAR implementer. In March of 2012, RHSP introduced the PIMA POC CD4 platform into the clinical program to expedite assessment of ART eligibility and monitoring of care. This evaluation consists of POC testing done until May 2012 among HIV-infected patients who were both ART naïve (pre-ART) and ART experienced persons. No informed consent was required as data was originally collected for clinical purposes and was analyzed anonymously.

The study was approved by institutional review boards at the Uganda Virus Research Institute's Safety and Ethics Committee, the Uganda National Council for Science and Technology, and Johns Hopkins University, School of Medicine.

### POC CD4 Testing

POC CD4 testing was performed with the PIMA CD4 analyzer. All testing followed manufacturer's instructions and involved use of four PIMA machines. The machines were located at a testing area next to the clinics and the venous samples were collected by the nursing team. Briefly, prior to daily testing, normal and low controls were run on the PIMA analyzer. Test cartridges were inspected for expiry dates, assembled, and participant identifiers entered [9], [15]. All testing was carried out by qualified laboratory technicians who had received training in PIMA usage. A result print out was available via an external PIMA printer for clinical review. At the end of each day, an electronic record of all tests identifiers and results was extracted from the PIMA and entered into a clinical cohort database.

PIMA testing was conducted on venous blood collected in a sterile 4 mL EDTA tube and inverted 8–10 times. All samples analyzed on PIMA were stored at ambient temperature (18–25°C) and run within 8 hours of collection. The tube was further inverted 10–15 times to ensure adequate sample mixing prior to volumetric pipette transfer to fill the sample collector of the PIMA CD4 test cartridge. The remaining blood was stored in cool boxes and dispatched to the laboratory for flow cytometry.

### FACSCalibur Testing

CD4 flow cytometry was performed using FACSCalibur (Becton Dickinson, San Jose, California, USA). CD4 estimation was done per manufacturer's instructions by trained laboratory technicians. The FACSCaliburs are regularly serviced and run external quality proficiency samples from UKNEQAS leukocyte immunophenotyping and College of American Pathologists (CAP). Samples were kept at ambient temperature (20–25°C) and run on FACSCalibur within 24 hours of blood collection. Daily instrument calibration was done using calibrate beads to adjust settings, set fluorescence compensation and ensure instrument sensitivity. Sample preparation was done by adding 20 uL of Tritest CD3, 4, 45 antibodies to a patient labeled truecount tube and 50 uL of whole blood prior to 15 minutes dark room incubation after which samples were loaded and analyzed. Work lists were generated, saved and results examined with manual gating done to fine adjust the CD4population before review by the laboratory manager. Data was exported and merged with the clinical cohort database.

### Statistical Methods

Descriptive statistics were used to summarize patient characteristics. Accuracy of CD4 results (FACSCalibur versus PIMA) were compared using the Bland-Altman Method which assesses bias as measured by the average difference and calculated 95% limits of agreement (LOA) of all the paired measurements [16]. Paired t tests were done to compare differences in means. Two by two tables were constructed to assess the diagnostic accuracy at CD4 thresholds of <350 and <500 cells/uL thresholds. The <350 threshold is the current Ugandan criteria for ART initiation and the 500 threshold is the new WHO recommended criteria for initiation of ART. All data were analyzed with STATA 12.1 software (College Station, Texas, USA).

## Results

**Figure 1:**
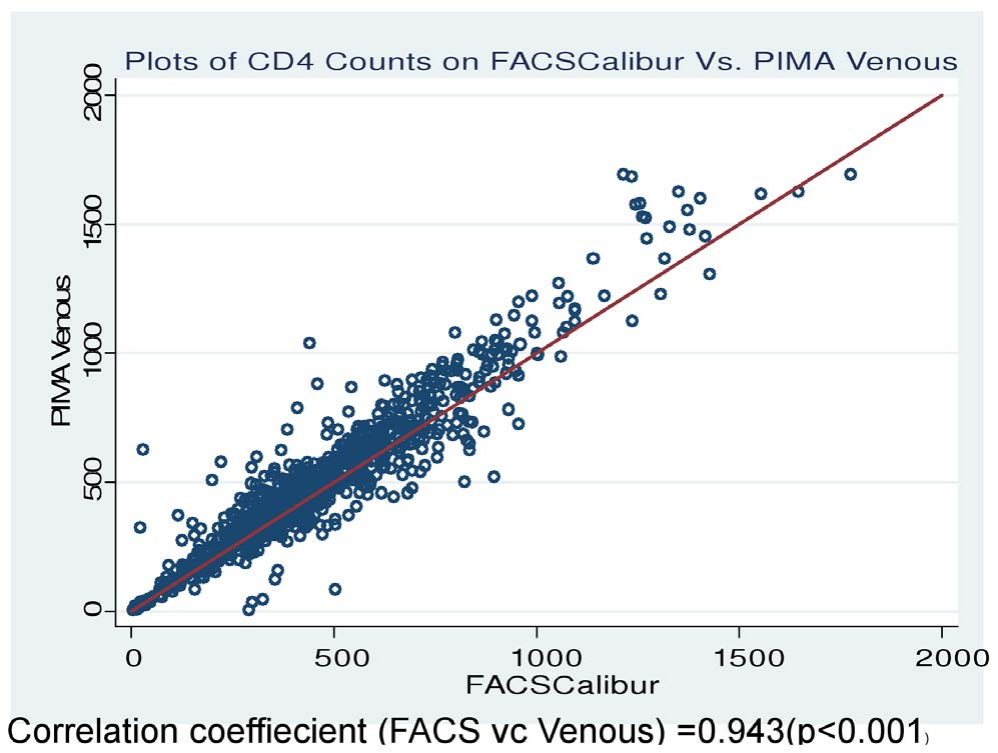
Scatter plots of FACSCalibur Vs PIMA measurements.

A total of 903 HIV-positive patients contributed data for this evaluation and 258 (28.5%) were on ART (Table 1). Scatter plots showed high correlation (r = 0.943, p<0.001) between PIMA and FACSCalibur estimates (Figure 1).

**Table 1:** Study population characteristics.

	Male	Female	Total
**Number of subjects (%)**	308 (34.1%)	595 (65.9%)	903
**Age, mean (range)**	39.9 (16 to 87)	36.4 (17 to 66)	37.6 (16 to 87)
**FACSCalibur, mean** CD4 cells/ul **(range)**	446.0 (5.2 to 1625.6)	537.0 (3.8 to 1691.8)	506.0 (3.8 to 1691.8)
**PIMA venous, mean** CD4 cells/ul **(range)**	409.1 (5 to 1646)	503.6 (22 to 1776)	471.3 (5 to 1776)

**Figure 2:**
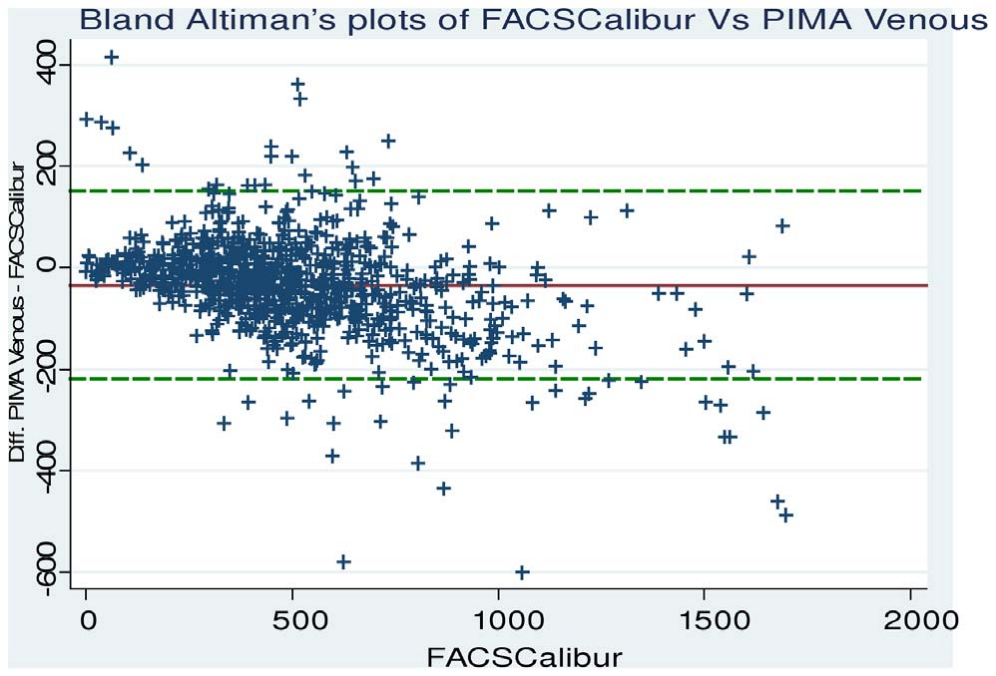
Bland-Altman plots comparing FACSCalibur and PIMA venous measurements.

Paired PIMA and FACSCalibur results were compared using Bland-Altman analysis (Fig. 2). The mean bias for the PIMA testing was −34.6 cells/µl (95% LOA: −219.8 to 150.6. Table 2). The overall mean CD4 count with PIMA was significantly lower than with FACSCalibur (p<0.0001), primarily due to underestimation of CD4 counts at higher CD4 levels ≥350 cells/uL (p<0.0001).

**Table 2:** Bias results of PIMA Venous versus FACSCalibur measurements.

	Total	<350	> = 350
**Number of subjects**	903	263	640
**Female sex (%)**	595 (65.9%)	151 (57.4%)	444 (69.4%)
**Age, mean (range)**	37.6 (16 to 87)	38.0 (16 to 87)	37.4 (17 to 87)
**FACSCalibur, mean (range)**	506.0 (3.8 to 1691.8)	224.2 (3.8 to 348.5)	621.8 (350.2 to 1691.8)
**PIMA venous, mean (range)**	471.3 (5 to 1776)	229.3 (5 to 503)	570.8 (30 to 1776)
**Absolute bias, cells/uL (SD)**	−34.6 (94.5)	5.1 (67.2)	−51.0 (99.2)
**LOA, mean (+/−1.96SD)**	−219.8 to 150.6	−126.6 to136.8	−245.4 to 143.4
**Paired t test for difference in means (95%CI;p)**	−34.6 (−40.8,−28.5; p<0.0001)	5.1(−3.1,13.2; p = 0.225)	−51.0 (−58.7,−43.3; p<0.0001)

The diagnostic accuracy of PIMA POC CD4 measurements was compared to FACSCalibur as the <350 cells/uL threshold for determining ART eligibility (Table 3). The PIMA sensitivity was 88.6% (95% CI 84.8–92.4%) and specificity was 87.5% (95% CI 84.9–90.1%).

**Table 3:** Sensitivity, Specificity, NPV and PPV at 350 cells/uL ART eligibility threshold.

Comparison measure	FACSCalibur vs PIMA venous, n/N (%) [95% CI]
**Sensitivity**	263/233 (88.6%) [84.8–92.4%]
**Specificty**	640/560 (87.5%) [84.9–90.1%]
**NPV**	590/560 (94.9%) [93.1–96.7%]
**PPV**	313/233 (74.4%) [69.6–79.2%]

We assessed the mean CD4 counts among patients who would be inaccurately classified as ART ineligible (i.e., false negative PIMA CD4 estimates) to better understand the likelihood of being found to be ART eligible at a subsequent test (Table 4). Samples misclassified by PIMA as above this level (350 cells/uL), had a PIMA mean CD4 count of 401 cells/uL which was significantly higher than the 350 cells/uL cut off (p<0.001). Similarly, samples misclassified by PIMA as false positive had a mean count of 307 cells/uL which was again significantly lower than the threshold of 350 cell/uL (p<0.001).

**Table 4:** CD4 Ranges by Instrument used at 350 cells/µl*.

FACSCalibur vs PIMA venous	mean cells/µl [range]	mean cells/µl [range]
**<350**	n = 233; 214.5 [3.76–348.51]‡ 207.1 [5–349]†	n = 80; 413.6 [350.2–627.4]‡ 307.0 [30–349]†
**≥350**	n = 30; 299.9 [85.2–346.6]‡ 401.3 [354–503]†	n = 560; 651.5 [350.3–1691.8]‡ 608.5 [351–1776]†

‡ FACSCalibur measurements mean and range.

† PIMA results.

Increasing the threshold 500 cells/uL significantly increased the PIMA sensitivity to 96.1% and PPV to 88.3%. However, specificity and NPV declined though not significantly (Tables 5 and 6). The PIMA mean CD4 count (566.2 cells/uL) of samples misclassified as negative (n = 20) was significantly higher than the cutoff of 500 cells/uL (p = 0.0001). Similarly, sample misclassified by PIMA as positive had an average PIMA CD4 count of 425 cells/µl which was significantly lower than the threshold of 500 cells/µl (p<0.0001, Table 7). At thresholds of 350 and 500, PIMA validity was higher in patients not on ART compared to patients on ART, though the differences were not statistically significant (results not shown).

**Table 5:** Bias results of PIMA Venous versus FACSCalibur measurements.

	Total	<500	> = 500
**Number of subjects**	903	516	387
**Female sex (%)**	595 (65.9%)	318 (61.6%)	277 (71.6%)
**Age, mean (range)**	37.6 (16 to 87)	38.2 (16 to 87)	36.7 (17 to 87)
**FACSCalibur, mean (range)**	506.0 (3.8 to 1691.8)	322.7 (3.8 to 499.9)	750.4 (500.5 to 1691.8)
**PIMA venous, mean (range)**	471.3 (5 to 1776)	311.8 (5 to 693)	684.1 (30 to 1776)
**Absolute bias, cells/uL (SD)**	−34.6 (94.5)	−10.9 (69.6)	−66.3 (112.4)
**LOA, mean (+/−1.96SD)**	−219.8 to 150.6	−147.3 to125.5	−286.6 to 154.0
**Paired t test for difference in means (95%CI;p)**	−34.6 (−40.8,−28.5; p<0.001)	−10.9 (−16.9,−4.9; p = 0.004)	−66.3 (−77.5,−55.0; p<0.001)

**Table 6:** Sensitivity, Specificity, NPV and PPV at 500 cells/uL ART eligibility threshold.

Comparison measure	FACSCalibur vs PIMA venous, n/N (%) [95% CI]
**Sensitivity**	516/496(96.1%) [94.4–97.8%]
**Specificty**	387/321 (83.0%) [79.2–86.7%]
**NPV**	341/321 (94.1%) [91.6–96.6%]
**PPV**	562/496 (88.3%) [85.6–91.0%]

**Table 7:** CD4 Ranges by Instrument used at 500 cells/µl*.

FACSCalibur vs PIMA venous	mean cells/µl [range]	mean cells/µl [range]
**<500**	n = 496; 318 [3.76–499.9]‡ 301.5 [5–496]†	n = 66; 565.2 [500.5–1037.3]‡ 425 [30–499]†
**≥500**	n = 20; 433.0 [85.2–499.0]‡ 566.2 [500–693]†	n = 321;788.5 [500.6–1691.8]‡ 737.3 [500–1776]†

‡ FACSCalibur measurements mean and range.

† PIMA results.

## Discussion

Delays in CD4 testing for assessment of ART eligibility is a significant impediment to ART rollout in LMIC. By reducing delays, POC technologies for CD4 estimation could help linkage into HIV care. We found that PIMA measurements compared to FACSCalibur produced negatively biased CD4 estimates which were more pronounced at CD4 counts >350 cells/µl. In our rural field setting, PIMA POC CD4 using venous blood demonstrated lower sensitivity at identifying persons eligible for ART at a CD4 count of 350 cells/µl. For the 11.4% of patients testing not eligible for ART by PIMA but eligible by FACSCalibur (i.e., false negatives), the mean PIMA estimates were significantly greater than 350 cells/uL, suggesting this patient population would likely benefit from early repeat testing. This finding is similar to a Kenyan study [17]. At a CD4 cutoff of 500 cells/uL, PIMA sensitivity increased significantly and this may support use of this technology when treatment guidelines recommend ART initiation at 500 cells/uL. Other field based studies are however necessary to establish the consistency of this finding.

POC CD4 testing with PIMA enables same day, on site immunological assessment of HIV positive clients due to its portability, printer options, limited blood volume requirements, and ease of use that eliminates initial blood processing and reagent preparation. In our setting, with a 20 minute turnaround time for each sample, busy clinics required two to four PIMA instruments and an additional technician to complete patient testing on any given day. This adds to cost in resource limited settings. A drawback identified by previous studies include inability of PIMA to predict CD4% for evaluations in children aged 2–6 years [12]. Our results showed increasing dispersion with CD4 counts >350 cells/uL which may reflect a proportional bias with increasing CD4 counts. It is noteworthy that in our study setting, the machines were moved to the testing site from the central laboratory everyday and this may have affected sensitivity and specificity for venous testing compared to a field evaluation in India [18]. The study in India was a multi-center study and each center had a PIMA instrument and a reference standard machine on site. In spite of this possibility, it is not known what an acceptable limit of misclassification should be and may well vary from program to program [12].

Limitations to this study include the fact that several technicians participated and there are possible inter-observer variations in machine handling. We also did not assess finger prick testing sampling in our study which may by an attractive option for some programs. Finally, our study was conducted at a single institution and results may not be generalizable to other settings.

Given the scarcity of FACSCalibur testing in many LMIC, POC technologies could facilitate screening for ART eligibility, but continued validation of POC CD4 platforms will be needed with scale-up in diverse situations.

## References

[1] Kagaayi J, Makumbi F, Nakigozi G, Wawer MJ, Gray RH, et al (2007) WHO HIV clinical staging or CD4 cell counts for antiretroviral therapy eligibility assessment? An evaluation in rural Rakai district, Uganda. AIDS 21 (9) 1208–10. 17502733 10.1097/QAD.0b013e32810c8dce

[2] Jaffar S, Birungi J, Grosskurth H, Amuron B, Namara G, et al (2008) Use of WHO clinical stage for assessing patient eligibility to antiretroviral therapy in a routine health service setting in Jinja, Uganda. AIDS Res Ther 5: 4. 18307778 10.1186/1742-6405-5-4 PMC2292208

[3] Lawn SD, Myer L, Orrell C, Bekker LG, Wood R, et al (2005) Early mortality among adults accessing a community-based antiretroviral service in South Africa: implications for programme design. AIDS 19 (18) 2141–8. 16284464 10.1097/01.aids.0000194802.89540.e1

[4] Lawn SD, Myer L, Harling G, Orrell C, Bekker LG, et al (2006) Determinants of mortality and nondeath losses from an antiretroviral treatment service in South Africa: implications for program evaluation. Clin Infect Dis 43 (6) 770–6. 16912954 10.1086/507095

[5] Brinkhof MW, Boulle A, Weigel R, Messou E, Mathers C, et al (2009) Mortality of HIV-infected patients starting antiretroviral therapy in sub-Saharan Africa: comparison with HIV-unrelated mortality. PLoS Med 6 (4) e1000066. 19399157 10.1371/journal.pmed.1000066 PMC2667633

[6] Stringer JS, Zulu I, Levy J, Stringer EM, Mwango A, et al (2006) Rapid scale-up of antiretroviral therapy at primary care sites in Zambia: feasibility and early outcomes. JAMA 296 (7) 782–93. 16905784 10.1001/jama.296.7.782

[7] Manabe YC, Wang Y, Elbireer A, Auerbach B, Castelnuovo B (2012) Evaluation of portable point-of-care CD4 counter with high sensitivity for detecting patients eligible for antiretroviral therapy. PLoS One 7 (4) e34319. 22536323 10.1371/journal.pone.0034319 PMC3334961

[8] Mnyani CN, McIntyre JA, Myer L (2012) The reliability of point-of-care CD4 testing in identifying HIV-infected pregnant women eligible for antiretroviral therapy. J Acquir Immune Defic Syndr 60 (3) 260–4. 22487589 10.1097/QAI.0b013e318256b651

[9] Diaw PA, Daneau G, Coly AA, Ndiaye BP, Wade D, et al Multisite evaluation of a point-of-care instrument for CD4(+) T-cell enumeration using venous and finger-prick blood: the PIMA CD4. J Acquir Immune Defic Syndr 58 (4) e103–11. 21909029 10.1097/QAI.0b013e318235b378

[10] Herbert S, Edwards S, Carrick G, Copas A, Sandford C, et al (2012) Evaluation of PIMA point-of-care CD4 testing in a large UK HIV service. Sex Transm Infect 88 (6) 413–7. 22544309 10.1136/sextrans-2012-050507

[11] Jani IV, Sitoe NE, Chongo PL, Alfai ER, Quevedo JI, et al (2011) Accurate CD4 T-cell enumeration and antiretroviral drug toxicity monitoring in primary healthcare clinics using point-of-care testing. AIDS 25 (6) 807–12. 21378535 10.1097/QAD.0b013e328344f424

[12] Mtapuri-Zinyowera S, Chideme M, Mangwanya D, Mugurungi O, Gudukeya S, et al (2010) Evaluation of the PIMA point-of-care CD4 analyzer in VCT clinics in Zimbabwe. J Acquir Immune Defic Syndr 55 (1) 1–7. 20622679 10.1097/QAI.0b013e3181e93071

[13] Glencross DK, Coetzee LM, Faal M, Masango M, Stevens WS, et al (2012) Performance evaluation of the Pima point-of-care CD4 analyser using capillary blood sampling in field tests in South Africa. J Int AIDS Soc 15 (1) 3. 22284546 10.1186/1758-2652-15-3 PMC3310849

[14] Kiwanuka N, Robb M, Kigozi G, Birx D, Philips J, et al (2004) Knowledge about vaccines and willingness to participate in preventive HIV vaccine trials: a population-based study, Rakai, Uganda. J Acquir Immune Defic Syndr 36 (2) 721–5. 15167291 10.1097/00126334-200406010-00009

[15] Alere website, Alere Pima CD4 Customer Training - http://www.alere.co.uk/sexual-health/articles/alere-pimatm-cd4-customer-training-241/text.htm, Alere.Accessed 2013 October 12.

[16] Bland JM, Altman DG (1986) Statistical methods for assessing agreement between two methods of clinical measurement. Lancet 1 (8476) 307–10. 2868172

[17] Mwau M, Adungo F, Kadima S, Njagi E, Kirwaye C, et al (2013) Evaluation of PIMA(R) point of care technology for CD4 T cell enumeration in Kenya. PLoS One 8 (6) e67612. 23825674 10.1371/journal.pone.0067612 PMC3692483

[18] Thakar M, Mahajan B, Shaikh N, Bagwan S, Sane S, et al (2012) Utility of the point of care CD4 analyzer, PIMA, to enumerate CD4 counts in the field settings in India. AIDS Res Ther 9 (1) 26. 22998738 10.1186/1742-6405-9-26 PMC3503578

